# Abrogation of contaminating RNA activity in HIV-1 Gag VLPs

**DOI:** 10.1186/1743-422X-8-462

**Published:** 2011-10-06

**Authors:** Ziyaad Valley-Omar, Ann E Meyers, Enid G Shephard, Anna-Lise Williamson, Edward P Rybicki

**Affiliations:** 1 Department of Molecular and Cell Biology, Faculty of Science, University of Cape Town, University Ave, Rondebosch 7701, South Africa; 2 Institute of Infectious Diseases and Molecular Medicine, Faculty of Health Sciences, University of Cape Town, Anzio Rd, Observatory 7925, South Africa; 3 Medical Research Council (South Africa), Tygerberg 7505, South Africa; 4 Department of Medicine, Faculty of Health Sciences, University of Cape Town, Anzio Rd, Observatory 7925, South Africa; 5 National Health Laboratory Service, Groote Schuur Hospital, Main Rd, Observatory 7925, South Africa

## Abstract

**Background:**

HIV-1 Gag virus like particles (VLPs) used as candidate vaccines are regarded as inert particles as they contain no replicative nucleic acid, although they do encapsidate cellular RNAs. During HIV-1 Gag VLP production in baculovirus-based expression systems, VLPs incorporate the baculovirus Gp64 envelope glycoprotein, which facilitates their entry into mammalian cells. This suggests that HIV-1 Gag VLPs produced using this system facilitate uptake and subsequent expression of encapsidated RNA in mammalian cells - an unfavourable characteristic for a vaccine.

**Methods:**

HIV-1 Gag VLPs encapsidating reporter **chloramphenicol acetyl transferase **(CAT) RNA, were made in insect cells using the baculovirus expression system. The presence of Gp64 on the VLPs was verified by western blotting and RT-PCR used to detect and quantitate encapsidated CAT RNA. VLP samples were heated to inactivate CAT RNA. Unheated and heated VLPs incubated with selected mammalian cell lines and cell lysates tested for the presence of CAT protein by ELISA. Mice were inoculated with heated and unheated VLPs using a DNA prime VLP boost regimen.

**Results:**

HIV-1 Gag VLPs produced had significantly high levels of Gp64 (~1650 Gp64 molecules/VLP) on their surfaces. The amount of encapsidated CAT RNA/μg Gag VLPs ranged between 0.1 to 7 ng. CAT protein was detected in 3 of the 4 mammalian cell lines incubated with VLPs. Incubation with heated VLPs resulted in BHK-21 and HeLa cell lysates showing reduced CAT protein levels compared with unheated VLPs and HEK-293 cells. Mice inoculated with a DNA prime VLP boost regimen developed Gag CD8 and CD4 T cell responses to GagCAT VLPs which also boosted a primary DNA response. Heating VLPs did not abrogate these immune responses but enhanced the Gag CD4 T cell responses by two-fold.

**Conclusions:**

Baculovirus-produced HIV-1 Gag VLPs encapsidating CAT RNA were taken up by selected mammalian cell lines. The presence of CAT protein indicates that encapsidated RNA was expressed in the mammalian cells. Heat-treatment of the VLPs altered the ability of protein to be expressed in some cell lines tested but did not affect the ability of the VLPs to stimulate an immune response when inoculated into mice.

## Background

Inert virus-like particles (VLPs) made from virus structural protein(s) are an ideal substitute for live, attenuated and peptide-based virus vaccines, as they present epitopes in an immunologically relevant context and lack any replicative nucleic acid. Several VLP-based vaccines have been developed against human viruses, such as the recently-released human papillomavirus (HPV) vaccines, and the well-established hepatitis B virus (HBV) vaccines [1-3]. Gag polyproteins of human and simian immunodeficiency viruses (HIV and SIV) produced in various expression systems (bacterial, yeast, insect, mammalian and plant cells) also assemble into VLPs that bud through plasma membranes to produce enveloped particles, which strongly resemble immature virions. These VLPs have been shown to be potent stimulators of both cellular and humoral immune responses in animal models and therefore are potentially excellent vaccine candidates [4-11].

During virion assembly, HIV Gag encapsidates two copies of the viral genomic RNA displaying a HIV packaging signal called the ψ-site [12-17]. The incorporation of specific viral RNA, though, is not a prerequisite for virion assembly and release [18-23]. In the absence of ψ-site-containing viral RNA, assembling viral particles still encapsidate high levels of host cell derived RNA [13,21,21,22,24]. This non-specific RNA encapsidation is mediated by RNA binding to basic residues distributed throughout various domains of Gag [18,19,23,25-28]. Gag proteins with specific mutations in these domains were shown to be unable to package RNA, and consequently unable to assemble into particles [18,23]. RNA has thus been shown to play a key structural role in virus particle assembly by serving as a scaffold upon which multiple Gag molecules can assemble [19,23,29-33]. As a result, HIV Gag VLPs produced in the aforementioned expression systems may thus contain significant levels of encapsidated host cellular RNA.

The primary mode of HIV entry into cells was assumed to be by Gp120/Gp41-and CD4+ host cell CD4 + CCR5/CXCR4 receptor-mediated plasma membrane fusion [34,35]. More recent studies have documented viral entry into cells mainly via a Gp120/Gp41-independent endocytic pathway, with HIV particles shown to be present within acidified endocytic vesicles destined for degradation [36-41]. At least 50-90% of viral material has been shown to enter cells by this Gp120/Gp41-independent endosomal pathway when viral particles contain functional Gp120/Gp41 [36-38,41], which suggests that the endocytic/degradative pathway is a major entry route. HIV virions may also be internalised by macropinocytosis in a receptor and pH-independent process [39,41]; however, only Gp120/Gp41-displaying virions were shown to access the cytosol, presumably by engaging specific receptors on the internal surface of the macropinosome/endosome. The general consensus of HIV cellular entry studies is that, irrespective of the path of entry into the target cell, successful HIV infection is dependent on receptor/co-receptor interactions at the cell surface or within vesicles.

HIV-1 Gag VLPs are often produced in baculovirus-based insect cell expression systems, which provide an ideal eukaryotic environment for the production of biologically active recombinant proteins as they enable post-translational modifications such as proper protein folding, disulphide bond formation, oligomerisation and proteolytic cleavage [42]. VLPs that bud from insect cells accordingly become enveloped with the insect host cell membrane, which contains abundant baculovirus Gp64 envelope glycoprotein. These VLPs are said to be pseudotyped with Gp64. Gp64 is one of the most abundant proteins comprising the baculovirus virion, and is crucial for infectivity and functionality of the baculovirus expression system [43]. Gp64 mediates baculovirus entry into cells via an influenza-like, clathrin-mediated, low pH-dependent endocytic pathway, where viral and endosomal membrane fusion occurs subsequent to endosomal acidification [44-53].

Gp64-mediated baculovirus uptake is not limited to insect cells. Several studies have demonstrated the ability of recombinant baculoviruses to enter host mammalian cells, and subsequent gene expression to occur within these cells. Their uptake has been demonstrated *in vivo *in mouse nasal epithelia, mouse skeletal muscle cells, rabbit endothelial cells, and *in vitro *in various mammalian cell lines including HeLa, Vero, BHK-21, 293 T-cells, rat hepatocytes and human hemocytes and hepatocytes [54-61]. Lentiviral (HIV/MLV) vectors pseudotyped with Gp64 have been shown to be taken up by mammalian cell lines with the same target cell range as that observed for recombinant baculoviruses, which include cells of hepatic origin, 293 T-cells, HeLa and HuH-7 human cell lines, while haematopoietic cells were not [62-64]. In addition, Schauber et al [63] and Sinn et al [64] have demonstrated the efficient gene transfer potential of Gp64 pseudotyped lentiviral (HIV/FIV) vectors *in vivo*.

While endosomal degradation of non-pseudotyped (Gp160) VLPs subsequent to cellular entry should effectively prevent the transmission and expression of VLP-contained RNAs, the presence of baculovirus Gp64 on the surface of Gag VLPs could enable expression of these RNAs within eventual mammalian vaccine recipients. The potential delivery and expression of insect cell-derived nucleic acids by VLPs within a vaccinated individual is regarded as a problematic trait by vaccine regulatory bodies such as the United States Food and Drug Administration (FDA). The FDA specifically requires vaccine developers to show that VLPs do not encapsidate "specific" nucleic acid sequences from the expression system, and especially those encoding VLPs components.

In this study we tested the ability of insect cell-produced HIV Gag VLPs to function as a vector for RNA uptake in several mammalian cell lines. Also, as the amount of Gp64 on the surface of insect cell-produced HIV-1 Gag VLPs has not been determined, this study initially estimated the relative levels of Gp64 per VLP. This study is the first, to our knowledge, to analyse the potential transfer and successful expression of encapsidated reporter CAT RNAs from non-replicating Gag VLPs in mammalian cell lines: previous studies have all been conducted on more complex live lentivirus models that carried reporter genes as part of the replicative viral genome [62,63]. VLPs were heated in an attempt to inactivate any RNA encapsidated by the VLPs and the CAT expression in mammalian cells after uptake was investigated. In addition, we compared the ability of heat-treated VLPs and unheated VLPs to stimulate an immune response in mice. GagCAT VLPs were immunogenic in mice, and heating of the VLPs appears to enhance the immunogenicity of these VLPs especially when used as a booster vaccine after a prime with a matching DNA vaccine.

## Methods

### Baculovirus-produced GagCATVLPs

**Figure 1:**
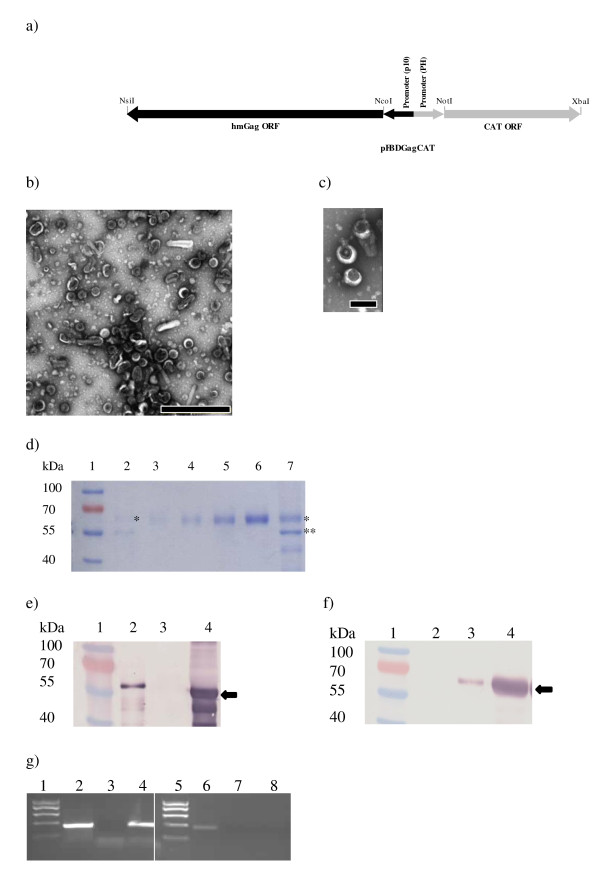
**VLP isolation and quantification**. a) Schematic diagram of pFBDGagCAT showing the Gag and CAT open reading frames under the control of the p10 and pH promoters, respectively. (b) Transmission electron micrograph of purified GagCAT VLPs. Arrows indicate typical doughnut-shaped VLPs (120 - 150 nm in diameter). Scale bar = 1000 nm. (c) Transmission electron micrographs of Gag VLPs after heating at 62°C for 20 minutes. Scale bar = 200 nm. (d) Coomassie-stained 10% SDS polyacrylamide gel used to densitometrically quantify Gp64 on in Gag VLP preparations. Lane 2 contains the Gp64 positive control (*), while lanes 3-6 contain dilutions of BSA at 100, 200, 500 and 1000 ng, respectively. Lane 7 contains Gag VLPs containing 1000 ng of Gag, showing gp64 (*) and Gag^Pr55 ^(**). (e) and (f) Comparative western blots of purified VLP extracts probed with anti-Gag primary antibody (e) and anti-Gp64 primary antibody (f): Lane 2 - HIV-1 p17/p24 C clade protein standard (ARP695.2); Lane 3 - Gp64 positive control; Lane 4 - Purified GagCAT VLPs. Black arrows indicate the position of the 55 kDa HIV Gag (e) and 64 kDa VSV Gp64 (f) proteins, respectively. (g) 1% agarose gel showing DNA fragments generated by RT-PCR (lanes 2-4) and PCR (lanes 6-8) of GagCAT VLPs using CAT-specific primers. Lane 1 - molecular weight marker; Lanes 2 and 6 - 350bp positive control fragments generated using *in vitro*-transcribed CAT RNA and CAT DNA for RT-PCR and PCR, respectively; Lanes 3 and 7 - result of negative control experiments generated by RT-PCR and PCR of Gag VLP RNA extracts, respectively; Lanes 4 and 8 - results of RT-PCR and PCR using GagCAT VLP RNA extracts, respectively.

A Pr55 *gag *gene encoding the Gag sequence with a myristoylation signal, derived from the Du_422 _clinical isolate [65,66] was human codon-optimised (hmGag; Operon Technologies (USA)) and cloned into the pFastBac™ Dual (pFBD) baculovirus insect cell expression vector (Invitrogen) under the p10 promoter to produce the pFBDGag construct. The pFBDGagCAT construct was generated by cloning a chloramphenicol acetyltransferase (CAT) reporter gene, derived from the pcDNA3.1/Zeo/CAT cloning vector (Invitrogen), into the gag-containing pFastBac™ Dual construct under the polyhedrin (PH) promoter (Figure 1a). Baculovirus produced pFBDGagCAT and control pFBDGag bacmids were generated in a *Spodoptera frugiperda*-derived *Sf*21 cell line (Invitrogen) [7,42,67-70]. Briefly, the *Sf*21 cells were maintained as a monolayer at 27°C in TC-100 insect medium (Sigma) supplemented with 10% (v/v) foetal bovine serum (FBS), 50 μg/ml neomycin, 69.2 μg/ml penicillin G and 100 μg/ml streptomycin. *Sf*21 cells (1 × 10^6 ^cells/ml) were infected with the recombinant pFBDGagCAT and control pFBDGag baculovirus at a multiplicity of infection (MOI) of 2-10, and VLPs were harvested from 100 ml of culture supernatant 72 h post infection by means of cross-flow filtration using a cross-flow filter unit (MidGee™, GE Healthcare) with a 300 kDa cut-off filter cartridge according to the manufacturer's protocol. VLPs were pelleted by centrifugation at 83, 000 × *g *and resuspended in 300 μl of 1 × sterile Dulbecco's phosphate buffered saline (PBS, Sigma^®^) and incubated with 7.5 u RNase A and 1.33 u RNase T1 for 2 h at 37°C [23] to remove any RNA in the surrounding medium. VLPs were re-purified by centrifugation through a 20% sucrose cushion at 83, 000 × *g *and resuspended in PBS containing 15% trehalose to maintain VLP stability.

### Electron microscopy of GagCAT VLPs

GagCAT VLPs were visualized using a LEO 912 transmission electron microscope after adsorption onto 200 mesh carbon-coated copper grids stained with 2% uranyl acetate.

### Quantification of Gag and Gp64 of GagCAT VLPs

The purity of VLPs were assessed and Gag concentration of the GagCAT VLP preparation was measured by electrophoresis of purified VLPs through a 10% denaturing SDS polyacrylamide gel and western blotting [68]. To quantitate the Gag concentration a serial dilution of an HIV-1 p17/24 C clade protein standard (ARP695.2, FIT Biotech, Programme EVA, centre for AIDS reagents, NIBSC) was included on the gel. Membranes were probed with a 1/5000 dilution of anti-p24 polyclonal rabbit antiserum (ARP432, NISBC Centralised Facility for AIDS reagents, MRC, UK) and a 1/5000 dilution of an anti-rabbit alkaline phosphatase-conjugated secondary antibody (Sigma-Aldrich). Gag protein was visualised using 5-bromo-4-chloro-3-indolyl phosphate/nitro blue tetrazolium (BCIP/NBT) (Roche). The Gag concentration of the GagCAT VLP preparation was estimated by densitometry which compared the collective density of all Gag bands with the density of the Gag bands for the standard using gel imaging software (Gene Genius Bio, Syngene). The VLPs were resuspended at 1 μg Gag/μl PBS containing 15% trehalose and stored at -70°C. The Gp64 content of the GagCAT VLP preparation was visualized by probing the membrane with a 1/5000 dilution of a purified mouse anti-baculovirus Gp64 envelope glycoprotein antibody (Clone: AcV5; eBioscience) and anti-mouse alkaline phosphatase-conjugated secondary antibody (Sigma-Aldrich) and developed with BCIP/NBT. Gp64 was quantitated from Coomassie-stained gels using serial dilutions of a BSA standard and densitometry as described for quantification of Gag protein above and variance was determined using Prism 5 (GraphPad).

### Inactivation of VLP RNA

For use in cell-uptake experiments, GagCAT VLP samples were heated at 62°C for 20 minutes (called hVLPs) based on the method used for heat-inactivating live HIV particles [71].

### Detection and quantitation of CAT RNA

RT-PCR (Access RT-PCR System, Promega) of GagCAT VLPs and these VLPs after heat treatment (hVLPs) contained 5 μl of the VLP preparation (equivalent to 5 μg Gag protein) in 100 μl and 1 mM MgCl_2 _with 50 pmol of the primers designed to yield a 350 bp product. The forward and reverse primer sequences used were 5' GCAATGAAAGACGGTGAGC 3' and 5' ATGAACCTGAATCGCCAGC 3', respectively. *In vitro*-transcribed CAT RNA was used as a positive control (Ribomax™ Large Scale RNA Production System - T7 kit (Promega). The cycle profile was as follows: 45°C, 45 min; 94°C, 2 min; 25 cycles of 94°C, 30 sec; 57°C, 30 sec; 72°C, 1 min.

RNA was extracted from GagCAT VLPs after heat treatment using the RTP Virus DNA-RNA Isolation Kit (Invitek). Each RNA extraction was carried out using 5 μl of the VLP preparation (equivalent to 5 μg Gag protein) and the RNA was eluted in 50 μl of extraction kit elution buffer after which 10 μl was used for real-time RT-PCR. These reactions were performed using a SensiMix One-Step real-time RT-PCR kit (Quantace) and a Rotor-Gene RG-3000A real-time PCR machine (Corbett Research). Reactions (25 μl) contained *in vitro*-transcribed CAT RNA as a standard or 10 μl of the RNA extracted from the GagCAT VLP preparation, 50 mM MgCl_2 _and 50 pmol of forward and reverse primers to amplify a 150 bp DNA fragment. The forward and reverse primer sequences used were 5' AGATGTGGCGTGTTACGGT 3' and 5' ATGAACCTGAATCGCCAGC 3', respectively. The reaction profile used was as follows: 49°C, 45 min; 95°C, 10 min; 30 cycles of 95°C, 15 sec, 57°C, 15 sec and 72°C, 15 sec. All real-time RT-PCR data was analysed using the Rotor-gene 6, Version 6.0 (Build 27) software (Corbett Research).

### GagCAT VLP uptake by mammalian cell lines

Murine macrophages (RAW 264.7), baby hamster kidney cells (BHK-21), human embryonic kidney cells (HEK 293) and HeLa cells were maintained as continuous cultures at 37°C in 5% CO_2_. RAW 264.7 cells were cultured in RPMI (RPMI 1640+GlutaMAX™-1, Gibco^®^) and BHK-21 cells, HEK 293 and HeLa cells were cultured in DMEM (DMEM+GlutaMAX™-1, Gibco^®^). These media were supplemented with 10% (v/v) foetal calf serum (Gibco), 1% penicillin G/streptomycin and 1% Fungin (supplemented culture medium). Twenty four hours prior to GagCAT VLP uptake the RAW 264.7, BHK-21 and HeLa cell cultures were seeded in 6-well plates (Nunc™) in 2 ml supplemented culture medium at a concentration of 0.5 × 10^5 ^cells/ml per well, while HEK 293 cells were seeded in 6-well plates at 0.2 × 10^5 ^cells/ml supplemented culture medium per well. Cells were washed twice with 2 ml PBS prior to the addition of GagCAT VLPs in a volume of 1 ml unsupplemented culture medium and incubated at 37°C for 2 h, which was then replaced with 2 ml of supplemented culture medium and incubated for 24 h at 37°C after which cell lysates were prepared and CAT protein content analysed by ELISA (Roche CAT ELISA kit). Each cell-type transfection was carried out with the same VLP and heated VLP preparations and the entire experiment was carried out in triplicate. ELISA plates were analysed using a PowerWave™ XS ELISA plate reader (BioTek^®^) and results analysed using the KCL™ (version 3.4) program (BioTek^®^). Mean values of triplicate assays for a cell lysate did not vary by more than 5%. Data is presented as mean responses for 6 replicate uptake experiments.

### Immune responses in mice

Female BALB/c mice (8-10 weeks old) were used to compare immune responses of GagCAT VLPs and heated GagCAT VLPs (hGagCAT VLPs) when given alone. The ability of these VLPs to boost a primary response induced by a matched HIV-1 subtype C DNA vaccine, pTHGagC [7] was also determined. All mouse procedures were approved by the UCT Animal Ethics Committee, (AEC no. 006-007). Plasmid (100 μg DNA) and VLPs (400 ng Gag protein) in PBS were given as intramuscular injections in a final volume of 100 μl with 50 μl injected into each hind leg muscle. Spleens were collected from groups of mice on day 40 after a prime inoculation on day 0 with pTHGagC and a boost with either GagCAT VLPs, hGagCAT VLPs or pTHGagC on day 28. Groups of mice were also left unvaccinated and then on day 28 vaccinated with GagCAT VLPs or hGagCAT VLPs and spleens collected on day 40 to determine responses to these VLPs only [68]. Splenocyte suspensions were prepared from spleens pooled from 5 mice per group and red blood cells (RBC) were lysed (RBC lysing solution, Sigma). IFN-γ and IL-2 ELISPOT assays (BD Pharmingen) were performed according to manufacturer's instructions to assess the frequency of Gag-specific IFN-γ and IL-2 secreting CD8 and CD4 T cells. Triplicate wells contained 500 000 splenocytes/well in a final volume of 200 μl R10 medium (RPMI 1640+GlutaMAX™-1 with 20 mM HEPES, 10% heat inactivated FCS, 15 mM β-mercaptoethanol, 1% penicillin G/streptomycin) only to determine background responses or in this medium with the peptides (> 95% pure; Bachem, Switzerland) AMQMLKDTI (Gag CD8) or NPPIPVGRIYKRWIILGLNK (Gag CD4(13) peptide) or FRDYVDRFFKTLRAEQATQE (Gag CD4(17) peptide) [72,73]. Spots were detected using Nova Red substrate (Vector Labs) then scanned and counted using a CTL Analyzer (Cellular Technology, OH, USA) with Immunospot Version 3.2 software. The mean number of spots from triplicate wells ± SD (standard deviation) was calculated and mean background spots were subtracted then adjusted to spot forming units (sfu) per 10^6 ^splenocytes ± SD.

## Results and discussion

### Characteristics of baculovirus-produced GagCAT VLPs

Transfection of *Sf*21 insect cells with pFBDGagCAT yielded VLPs that could be harvested by cross-flow filtration from insect cell culture supernatant. Transmission electron microscopy of the purified VLPs showed numerous doughnut-shaped particles with diameters ranging in size from 120 to 150 nm (Figure 1b). A few baculovirus particles were occasionally observed in samples of the VLP preparations viewed under the electron microscope, but not in all fields of view. Although the ratio of baculovirus particles to VLPs was not calculated, it was considered too small to interfere with the estimated calculation of Gp64 protein per μg of Gag protein by much. A Coomassie-stained gel of GagCAT VLP preparations showed a dominant 55 kDa band of HIV-1 Gag protein (Figure 1d - lane 7**). The higher band is thought to be the 64 kDa gp64 protein and the lower band is a p41 HIV-1 Gag breakdown product which has been observed in previous work in this laboratory [67]. Western blotting of GagCAT VLPs using an anti-Gag antibody confirmed the presence of the 55 kDa HIV-1 Gag protein (Figure 1e - lane 4). A western blot of GagCAT VLPs using an anti-Gp64 antibody showed a band of 64 kDa in size (Figure 1f - lane 4) which confirmed the association of this protein with the purified GagCAT VLP preparations. As there was no quantified Gp64 standard available for western blot or ELISA quantitation, the relative amount of Gp64 incorporated into the GagCAT VLPs per microgram of VLPs Gag protein was determined densitometrically using quantitated amounts of BSA on a Coomassie-stained gel (Figure 1d - lanes 3 to 6). The average Gp64 concentration was calculated to be 383 ± 86 ng Gp64/ug of Gag after analysis of four separate VLP purifications analysed in triplicate.

### CAT RNA in GagCAT VLPs

To determine whether CAT RNA was encapsidated in the purified GagCAT VLPs, RT-PCR using CAT-specific primers was carried out on purified GagCAT VLP RNA. The presence of CAT RNA was shown by successful amplification of a 350 bp DNA fragment (Figure 1g - lane 4), corresponding to the positive control, which was generated using *in vitro*-transcribed CAT RNA (Figure 1g - lane 2).

No product was amplified from RNA extracted from Gag VLPs (Figure 1e - lane 3 and 7) which were produced after transfection of *Sf*21 insect cells with a construct lacking CAT (pFBDGag). To exclude the possibility that DNA fragments could have resulted from amplification of contaminating CAT DNA co-purified from the insect-cell preparation, GagCAT VLPs were subjected to PCR using CAT-specific primers, which failed to amplify any product (Figure 1g - lane 8). A 350 bp DNA fragment was observed from amplification of CAT DNA (lane 6) and no fragment was observed from amplification of Gag VLP RNA (lane 7), as expected.

The amount of CAT RNA encapsidated by 1 μg of Gag VLPs was quantitated by real-time RT-PCR using *in vitro*-transcribed RNA as a standard. CAT RNA quantitation of nine individual GagCAT VLP preparations showed that the amount of encapsidated CAT RNA/μg Gag VLPs varied between individual VLP preparations (0.1 - 7 ng CAT RNA/μg Gag VLPs) (data not shown).

### Expression of encapsidated CAT RNA in mammalian cell lines

**Figure 2:**
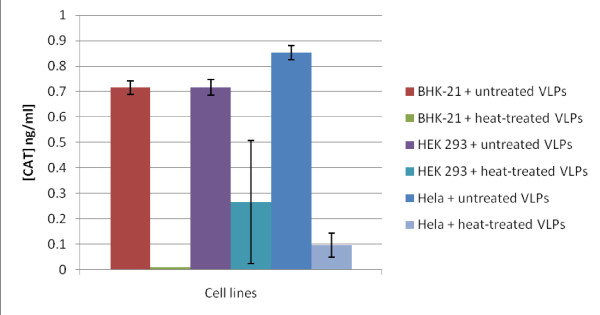
**Expression of encapsidated CAT RNA in mammalian cell lines**. CAT expression levels are shown in BHK-21 (I), HeLa (II) and HEK 293 (III) cell lysates 24 hours after unheated or heat treated VLP uptake. Gag VLPs containing 10 ng of CAT RNA were used for the cell uptake assays in each cell line. Heat treated VLPs were incubated for 20 min at 62°C. Error bars display the variance between 3 separate experiments. P values are displayed above each set of uptake assays.

To test whether CAT RNA could indeed be transcribed and translated into protein in mammalian cells, we determined the presence of CAT protein in BHK-21 cells after transfection with *in vitro*-transcribed CAT RNA. CAT RNA transcription and translation was confirmed by the detection of CAT in cell lysates (data not shown). To investigate whether CAT RNA can be expressed in mammalian cells after uptake of GagCAT VLPs, VLPs containing 10 ng of CAT RNA were added to 4 different mammalian cell types and CAT protein expression levels in cell lysates quantified by CAT ELISA after 24 hours. The cell lines were selected on the basis of availability in the laboratory, as well as the fact that at least 3 of them are known to facilitate entry of Gp64-pseudotyped viral particles by means of a Gp64 receptor that is present on their outer cell wall [56,58,74]. Cell lysates prepared from BHK-21, HEK 293 and HeLa cells contained CAT protein (Figure 2). In contrast, lysates of the murine macrophage cell line (RAW 264.7), a hematopoietic cell line, known to be non-receptive to baculovirus Gp64 mediated transfection [57] did not contain CAT protein after incubation with the GagCAT VLPs (data not shown). CAT protein expression levels between the 3 different cell lines ranged from 0.7 - 0.86 ng/ml of CAT, when transfected with VLPs containing a total 10 ng of CAT RNA respectively (Figure 2).

### Effect of heat treatment on expression of encapsidated CAT RNA in mammalian cell lines

The cellular uptake and subsequent transcription of GagCAT RNA encapsidated by the VLPs has implications for their use as a vaccine. It is important to limit transmission and expression of RNA encapsidated by VLPs to ensure no production of a protein that may cause ill effects when VLPs are administered as a vaccine. We tested whether heating the VLPs (using a heating method based on that used to inactivate HIV particles [71,75]) would impact on the GagCAT transcription and translation after VLP uptake in certain mammalian cells. Gag VLPs carrying 10 ng of CAT RNA were incubated at 62°C for 20 minutes prior to incubation with BHK-21, HEK 293 and HeLa cells. Twenty four hours following cell uptake, cell lysates were analysed for CAT expression by CAT ELISA (Figure 2). Lysates of BHK-21 and HeLa cells after uptake of heat treated VLPs contained significantly reduced (p < 0.0001) CAT protein levels compared to lysates from cells that had been incubated with unheated VLPs. On the contrary, expression of CAT in HEK-293 cells after uptake of heat treated VLPs was marginally different from that after uptake by unheated VLPs (Figure 2). Heat-treated VLPs were not physically or structurally damaged (Figure 1c). TEM proved them to be intact doughnut-shaped particles, similar to non-heated VLPs as shown in Figure 1b.

### GagCAT VLPs before and after heat treatment are immunogenic in mice

**Figure 3:**
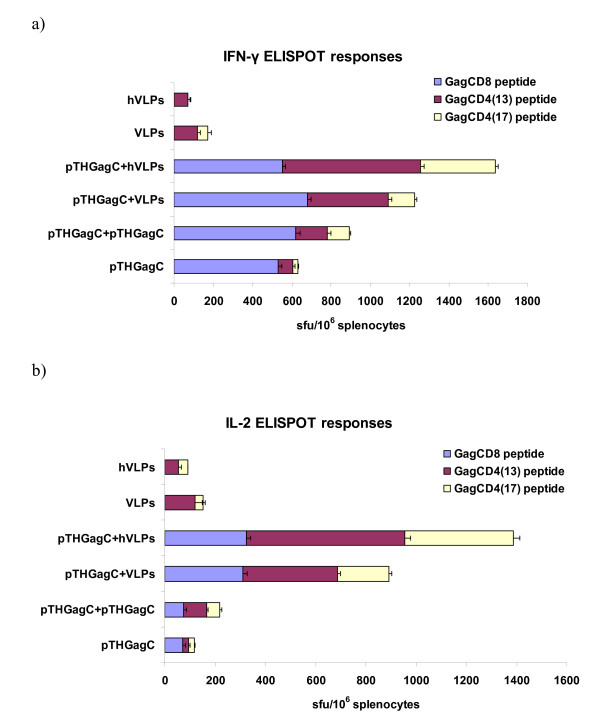
**Immune responses in mice vaccinated with GagCAT VLPs**. IFN-γ (A) and IL-2 (B) ELISPOT responses of groups of BALB/c mice to Gag CD8 and Gag CD4 peptides on day 12 after vaccination with GagCAT VLPs (VLP) or heated GagCAT VLPs (hVLP) or pTHGagC and on day 40 for mice primed with pTHgagC then boosted on day 28 with VLPs or hVLPs. Splenocytes prepared from spleens combined from five mice per group were used in IFN-γ or IL-2 ELISPOT assays with the indicated Gag peptides. Bars are the mean number of spots of triplicate reactions for 10^6 ^splenocytes with indicated standard deviation of the mean. Data is that from one of three replicate experiments.

Since the aim of this work is to develop a vaccine that does not transmit foreign RNAs but will maintain an immune response to Gag, immune responses to single vaccinations of GagCAT VLPs and heat treated GagCAT VLPs were compared in BALB/c mice. In addition, the effect of heating GagCAT VLPs on their ability to boost a DNA vaccine prime (pTHGagC) was compared to that of two vaccinations of pTHGagC. T cell responses to a Gag CD8 peptide and two Gag CD4 peptides (Gag CD4 (13) and Gag CD4 (17)) were measured using IFN-γ and IL-2 ELISPOT assays as the measure of the immune response (Figure 3a). A VLP only vaccination induced a cumulative response to the Gag CD4(13) and Gag CD4(17) peptides of 192 sfu/10^6 ^splenocytes in the IFN-γ ELISPOT assay while a heat treated GagCAT VLP only inoculation induced a low response of 70 sfu/10^6 ^splenocytes to the Gag CD4(13) peptide (Figure 3a). No response to the Gag CD8 peptide was induced by either VLP vaccine (Figure 3a). Both VLP preparations boosted a primary response to pTHGagC and the magnitudes of these responses were greater than the sum of the responses to the individual priming and boosting vaccines (Figure 3a). A cumulative response to the Gag CD8 and Gag CD4 peptides of 1225 sfu/10^6 ^splenocytes in the IFN-γ ELISPOT assay was measured when GagCAT VLPs were used as the boost while a cumulative response to these peptides of 1637 sfu/10^6 ^splenocytes was measured when heated GagCAT VLPs were the boost vaccine. For the prime with pTHGagC and boost with either VLP preparation Gag CD8 T cells contributed approximately 600 sfu/10^6 ^splenocytes to these cumulative responses (Figure 3a). The higher response achieved with the pTHGagC prime and heat treated GagCAT VLP boost as opposed to a GagCAT VLP boost was due to a greater frequency (2 fold) of responding Gag-specific CD4 T cells. These responses to a DNA prime and GagCAT VLP or heat-treated GagCAT VLP boost were of higher magnitude than two pTHGagC vaccinations which reached a cumulative response of 892 sfu/10^6 ^splenocytes. The increase in response to two DNA vaccinations is due to an increase in responding Gag-specific IFN-γ producing CD4 cells, without a boost of the Gag CD8 cells. In comparison for two DNA vaccinations CD4 T cells contribute 30% to the overall response while CD4 T cells contribute 46% and 66% to the overall response when the boost to a DNA prime is either GagCAT VLPs or heated GagCAT VLPs respectively (Figure 3a)

GagCAT VLP and heat-treated GagCAT VLP vaccinations also induced Gag CD4+ cells that produced IL-2 with cumulative CD4 T cell frequencies of 153 sfu/10^6 ^splenocytes and 93 sfu/10^6 ^splenocytes respectively (Figure 3b). The prime-boost vaccination regimens induced Gag CD8 and CD4 IL-2 producing T cells and the magnitude of these responses were greater than the sum of the responses to the individual priming and boosting vaccines (Figure 3b). The sum of IL-2 producing Gag CD8 and Gag CD4 T cells induced by pTHGagC were boosted by GagCAT VLPs and heat treated GagCAT VLPs to levels of 893 sfu/10^6 ^splenocytes and 1387 sfu/10^6 ^splenocytes respectively. CD8+ cells contributed similar frequencies of 310 sfu/10^6 ^splenocytes to these total responses (Figure 3b). A pTHGag prime and hGagCAT VLP boost induced IL-2 producing CD4+ T cells of 1061 sfu/10^6 ^splenocytes which is almost twice that induced by the pTHGagC prime and GagCAT VLP boost.

## Conclusions

GagCAT VLPs that budded into the culture medium of *sf*21 insect cells using the baculovirus expression system were pseudotyped with baculovirus Gp64 envelope glycoprotein. Electron microscopic examination of the GagCAT VLP preparation revealed that the cross-flow filtration technique used to isolate the VLPs from the culture supernatant successfully limited co-purification of baculovirus particles to very low levels. In addition CAT-encoding DNA carried by baculovirus particles in the GagCAT VLP preparation was not detected by CAT-specific PCR of the VLPs, which further supported very low baculovirus contamination (Figure 1g).

Although Gp64 pseudotyping has been documented [76], we now report that the Gp64 content of baculovirus-produced VLPs is very high relative to Gp120 on HIV particles (~383 ng Gp64 per 1 μg Gag). HIV VLPs of 120 to 150 nm in diameter are estimated to contain ~5000 Gag proteins [77]. Thus each VLP contains ~1600 Gp64 molecules, or approximately 1 Gp64 molecule for every 3 Gag molecules in each VLP. The number of glycoprotein envelope spikes on infectious HIV virions was originally estimated to be approximately 72 [78], while more recent studies have indicated this number to be 2 to 14 trimers [78-81], which is sufficient to mediate specific CD4+ T-cell entry. The far greater number of baculovirus Gp64 molecules found on baculovirus-produced VLPs means these could very probably mediate a baculovirus-specific mode and specificity of cellular entry.

Previous research has shown that HIV particles that lack envelope glycoproteins are most likely to be degraded in endocytic vesicles [39,41], indicating that the entry and successful replication of HIV in mammalian cells is facilitated by the presence of glycoproteins on the outer surface of the viral particle. The Gp64 pseudotyped GagCAT VLPs containing a known quantity of encapsidated CAT RNA were thus assayed in our uptake experiments using BHK-21, HEK 293 and HeLa cells which are known to be permissive to Gp64-mediated recombinant baculovirus entry [54-56,58,60,61,74]. GagCAT VLPs were assumed to be taken up by these cell lines as the encapsidated CAT reporter RNA was successfully translated to produce CAT protein. The CAT protein levels yielded were similar irrespective of the type of cell in which they were produced. The encapsidated CAT RNA in our VLPs is representative of other RNAs that are normally present in the VLP expression system. To our knowledge, these results are the first to document the Gp64-mediated uptake of VLPs made using an insect cell expression system into mammalian cells, and the subsequent expression of a reporter RNA species encapsidated in the VLPs. Our results suggest that there is a strong possibility that RNA which is randomly encapsidated during VLP formation could be successfully translated into protein once it has been taken up by mammalian host cells. This finding contradicts previous assertions that baculovirus/insect cell-produced HIV-Gag VLPs are non-"infectious" [76].

The VLP heat inactivation experiments showed that heat treatment at 62°C reduced CAT protein levels in lysates of two of the cell types tested (BHK-21 and HeLa) to almost undetectable levels (Figure 2), which is in agreement with results obtained using live virus particles [71,75]. The application of heat to the VLPs was primarily to "disrupt" the particle surface structure: electron microscopic analysis of heat-treated VLPs showed that heat treatment did not noticeably disrupt or lyse the VLPs (Figure 1c). Heating of *in vitro-*transcribed CAT RNA prior to transfection also had no effect on CAT expression in the tested BHK-21 cell line (not shown). We concluded from this that the inhibition of CAT expression was most likely as a result of Gp64 damage on the GagCAT VLP surface. In the HEK 293 cells, CAT protein levels were relatively unaffected when higher levels of encapsidated RNA were used in the cell uptake assay. It is possible this is a result of the HEK-293 cells employing an additional or alternate VLP cell uptake pathway to that used by the BHK-21 and HeLa cell lines. This variation in sensitivity to heat may be cause for concern considering that a mixed population of cell types may be present in the area of inoculation when used as a vaccine in mammalian subjects. UV irradiation of VLPs has also been tested in this laboratory, but this had little effect on abrogating or even decreasing GagCAT transcription and translation (data not shown).

With regard to particle immunogenicity, a literature review has suggested that pseudotyping Gag VLPs with functional/fusion competent envelope glycoproteins may be of paramount importance in stimulating both MHC-I and MHC-I Gag processing pathways [11,82-85]. It has been shown that VLPs that did not display HIV-1 Env were unable to induce immune responses that were stronger than a DNA vaccine that produced soluble polypeptides [86]. In addition, HIV VLPs displaying HIV-gp120 taken up by monocyte-derived dendritic cells (MDDC) [87] showed enhanced Th1- and Th2-cytokine production and were able to activate autologous naïve CD4^+ ^T-cells and drive them towards a Th-1 response [87]. Monkeys immunized with VSV-G pseudotyped SIV particles generated SIV-specific humoral and cellular immune responses, which significantly reduced peak viraemia levels of a challenge virus. In mice, VSV-G-pseudotyped HIV VLPs stimulated Gag-specific IgG and IgG1 antibody titres that were ~100 fold higher than non-pseudotyped VLPs. As a control, a fusion defective VSV-G was also displayed on the HIV VLPs and was not able to enhance the VLP immune response. Insect cell-derived HIV/SIV-derived VLPs that have no specific mammalian virus-derived envelope glycoprotein have been found, nevertheless, to efficiently stimulate the MHC-1-driven CTL responses as well as humoral immune responses, which may be a result of the presence of the baculovirus Gp64 envelope glycoprotein [5,7,9,85,87-95].

The cellular immune response is partly dependent on the successful entry of viruses into the cytoplasm of the target cell resulting in viral replication rather than viral particle degradation which stimulates the Th1 immune response. This kind of entry would be facilitated by receptor-mediated Gp64 interactions. If heat treatment disrupts Gp64 on the Gag VLPs, successful entry of VLPs into the target cell would not occur, thus preventing a Th1 immune response to be stimulated. However, we showed that VLP heat inactivation did not appear to affect induction of subsequent immune responses (Figure 3). GagCAT VLPs both post-heating and unheated, induced low Gag-specific CD4 IFN-γ and IL-2 producing cells in mice and both VLPs preparations boosted immune responses induced by a primary DNA vaccine. It is not clear why a more substantial boost of the Gag-specific CD4 T cell response was observed with the hGagCAT VLPs. However, the VLP preparation only vaccinations and DNA vaccine prime VLP boost vaccination regimens induced magnitudes of Gag-specific immune responses as that induced by Gag VLPs [7,68].

In summary, BEVS-produced HIV-1 Gag VLPs were shown to be able to transfer and express foreign expression system-derived RNAs in mammalian cell lines. Transfection of mammalian cells with heat-treated VLPs did not prevent RNA expression in all cell lines, but reduced the range of cell lines that the VLPs could enter and thus the range of cells into which RNA was taken up. These results highlight the need to control the nucleic acid content of assembled Gag particles notwithstanding their need for random RNA encapsidation, crucial for particle assembly. A possible alternative to limit RNA encapsidation and hence transmission may lie with creating chimeric gag particles that do not require RNA for assembly, as has been shown by Crist et al., 2009 [96]. Chimeric Gag particles in which the nucleocapsid domain had been replaced with a trimerizing leucine zipper-domain showed an ability to assemble in 293T-cells, which released particles that lacked any detectable RNA. Future studies should investigate the feasibility of producing these chimeric particles in insect cell lines and assess their immunogenicity. Furthermore, where RNA transmission is evident, the RNA uptake pathways should be verified through the use of specific cell pathway uptake inhibitors or antibodies specific for functional receptors on the virion surface, such as Gp64.

## Competing interests

The authors declare that they have no competing interests.

## Authors' contributions

ZV-O produced and characterised the VLPs, carried out the PCR and RT-PCR and tested expression of the RNA in cell lines and drafted the manuscript. ES and her group were responsible for carrying out the mouse experiments and performing the ELISPOT assays. AM and EPR were involved in supervision of the work, critically revising the manuscript for important intellectual content, and together with A-LW, conceived of the study, and participated in its design and coordination. All authors read and approved the final manuscript.
